# The impact of urban agglomeration planning on depression in older adults

**DOI:** 10.3389/fpubh.2024.1456729

**Published:** 2024-12-17

**Authors:** Ya Liu, Li Yan, Yujue Wang, Xiaotang Tang, Ming Gao, Jiayu Yang, Zuoyan Liu, Xiuying Hu

**Affiliations:** ^1^Innovation Center of Nursing Research and Nursing Key Laboratory of Sichuan Province, West China Hospital, Sichuan University/West China School of Nursing, Sichuan University, Chengdu, Sichuan, China; ^2^Department of Critical Care Medicine, West China Hospital, Sichuan University, Chengdu, Sichuan, China; ^3^China Academy of Engineering Physics, Mianyang, Sichuan, China; ^4^Sichuan Vocational College of Commerce, Chengdu, Sichuan, China; ^5^School of Economics, Sichuan University, Chengdu, Sichuan, China; ^6^School of Business, Chengdu University of Technology, Chengdu, Sichuan, China; ^7^Department of Rehabilitation Medical Center, West China Hospital, West China School of Nursing, Sichuan University, Chengdu, Sichuan, China

**Keywords:** urban agglomeration planning, older adults, depression, difference-in-differences model, CHARLS

## Abstract

**Introduction:**

The residential environment significantly impacts the mental health of older adults. Urban agglomeration planning, while fostering regional economic development, also influences the psychological well-being of this demographic.

**Methods:**

This study investigates the effects of urban agglomeration planning on depression levels in older adults, utilizing cohort data from the China Health and Retirement Longitudinal Study (CHARLS) and the multi-temporal double-difference-in-differences (DID) model.

**Results:**

Our findings reveal that urban agglomeration planning alleviates depression by enhancing green spaces, improving air quality, and advancing digital infrastructure development. Moreover, these benefits are particularly pronounced among older females, residents of Central and Western regions, and those with lower educational attainment.

**Conclusion:**

Based on our research findings, we recommend expediting the dissemination of urban agglomeration construction experiences to alleviate depression among older adults. In the implementation of policies, it is essential to consider objective conditions such as geographical location and educational level. The formulation of differentiated urban agglomeration planning to promote mental health among older adults.

## 1 Introduction

As the global demographic shifts toward an aging population and the promotion of the “healthy aging” strategy gains prominence, geriatric mental health has increasingly become a focal point of scholarly attention within the mental health domain (1). Notably, Late-Life Depression (LLD), often described as a “psychological killer,” ranks as a major challenge in mental health care, second only to Alzheimer’s disease. It has emerged as a prevalent mental disorder that poses a significant threat to the mental well-being of older adults. Depression manifests primarily as a psychological syndrome characterized by persistent low mood, pervasive pessimism, and anhedonia, often accompanied by various somatic symptoms including cardiovascular issues and endocrine metabolic disturbances (2), as well as other neurological dysfunctions (3).

According to World Health Organization (WHO) statistics, the global prevalence of depression stands at 4.4% (4), with higher rates observed among the senior population aged 60 and above. In China, for instance, the prevalence of depression among community-dwelling older adults exceeds 36.25% (5). LLD not only impairs physical and mental health but also significantly contributes to the incidence of suicide among older adults (6). As individuals age, the disease burden associated with depression escalates markedly, adversely affecting their quality of life, escalating healthcare costs, and imposing a substantial economic burden on society. Addressing depression among older adults and ensuring their enjoyment of a healthy later life are thus pivotal concerns in senior research and broader societal development. These concerns are integral to mental health fields and the broader realization of a global “healthy aging” strategy.

As the dominant form of social development today, cities play a key role in promoting economic growth and improving social infrastructure. However, continued urban expansion also poses many challenges to human health. On the one hand, increased industrial activity and traffic density have led to air pollution, noise pollution, and a reduction in green space, which pose serious threats to urban ecosystems and public health (7, 8). On the other hand, the spatial distribution of healthcare resources is imbalanced, with an over-concentration of facilities in some areas and a lack of resources in others (9). This makes it difficult for residents to access healthcare services in a timely manner, further exacerbating health inequalities. In addition, fast-paced, high-stress urban life exposes residents to social isolation and mental health problems. A lack of social support and community cohesion, especially for vulnerable groups such as the older adult, is more likely to lead to feelings of loneliness and psychological issues like depression (10). Mitigating the threats posed by rapid urban development to the older adult population has become a major issue in many developing economies.

To address these challenges, the 2030 Agenda for Sustainable Development emphasizes the need to build inclusive, safe, disaster-resilient, and sustainable cities, particularly by providing safe, accessible, and green public spaces for older persons (11). Meanwhile, China’s 13th Five-Year Plan explicitly proposes to promote the efficient development of urban agglomerations, focusing on people-centered urbanization (12). In China’s high-quality development policy, the planning of urban agglomerations plays a key role as an important initiative for environmental improvement and ecological restoration. In this context, it is important to understand the impact of urban agglomeration planning on the mental health of older adults. Therefore, this study aims to address the following questions: First, is there a correlation between the implementation of urban agglomeration and the depressive states of older adults during the same period? Second, what are the mechanisms behind this correlation? Third, is the impact of urban agglomeration on older adult’s depression universal?

## 2 Literature review

To comprehensively explore the impact of urban agglomeration planning on depression in the older adult, this study begins with a literature review of the factors influencing depression and the role of urban environments in the health of older adults. By examining the multiple causes of depression and revealing the specific vulnerabilities of older adults in biological, psychological, and social contexts, we lay a theoretical foundation for the subsequent analysis of these factors in an urban agglomeration setting. In addition, we explore how social support, housing quality, public facilities, and the accessibility of green spaces in urban environments affect the health of older people. This approach not only aids in understanding how urban agglomeration planning can alleviate geriatric depression by optimizing these factors but also highlights in-depth explorations and innovative perspectives at the intersection of urban planning and geriatric mental health.

### 2.1 Factors influencing depression in older adults

The onset and development of depression result from multifactorial interactions, encompassing biogenetic, environmental, and social factors. Biologically, research underscores a genetic predisposition to depression, individuals with a family history of the condition are notably more susceptible (13). Moreover, abnormalities in neurotransmitter levels—specifically serotonin and norepinephrine—Within the brain of older adult individuals have been closely linked to the onset of depression (14). Additionally, prevalent chronic conditions among older adults, such as cardiovascular diseases, diabetes, and Parkinson’s disease, are significant risk factors for the development of depression (15). Secondly, environmental factors play an important role. Biophilia is recognized as a deep connection between humans and the natural environment, and natural interactions promote the secretion of neurotransmitters such as dopamine and serotonin, which are known as “pleasure hormones” and play a key role in mood stabilization and the maintenance of mental health. Regular exposure to natural landscapes can enhance emotional states and reduce the incidence of depression (16). Studies have also shown that fine particulate matter (PM2.5) from air pollution is positively associated with the incidence of depression in older adults, potentially affecting mental health by triggering chronic inflammation and neurological damage (17). Thirdly, Social factors also play a crucial role, the quality of the living environment substantially affects older adults’ mental health, where conducive and adaptable surroundings can markedly enhance their life quality and psychological well-being. Emotional support from family and friends has been found to effectively mitigate feelings of loneliness and stress, thereby reducing depression risk (18). Furthermore, active social engagement can foster connections, enhance self-worth, and diminish feelings of isolation among older adults, serving as a preventive measure against depression (10). In addition, personal characteristics are also important factors that cannot be ignored. Certain cognitive distortions common among older adults, such as catastrophic thinking, overgeneralization, and dichotomous thinking, have been shown to exacerbate depressive symptoms (19). Personality traits such as high levels of self-criticism and perfectionism can further intensify feelings of low self-esteem and depression, particularly when personal goals remain unmet (20). These multifaceted factors collectively shape the complex etiological network of depression in older adults. Therefore, identifying and effectively addressing controllable environmental and social factors is not only important for improving the mental health and social well-being of the older population but also a critical task in the field of public health.

### 2.2 Urban impacts on health in older adults

Existing studies have often framed health issues within the context of urban ecology, particularly in economically vibrant regions like the Beijing-Tianjin-Hebei, the Yangtze River Delta, and the Pearl River Delta. Urbanization and socio-economic growth have significantly impacted these areas, leading to pronounced environmental issues such as air pollution, water pollution, and a noticeable reduction in green spaces. Specifically, the presence of PM2.5 and PM10 particulates in the air is linked to an increased risk of respiratory and cardiovascular diseases (21). Simultaneously, green spaces are considered a factor in health and well-being. The reduction of green spaces limits the areas available for physical activity, thereby increasing the risk of chronic diseases such as obesity, cardiovascular diseases, and diabetes (22). For older adults, the reduction in natural environments can exacerbate depressive states, as these settings play a crucial role in psychological relaxation and emotional stability (16). The rapid expansion of urban agglomerations influences residents’ choices regarding living areas and the quality of their environments. The development and structuring of transportation infrastructure also reshape daily commutes and activity scopes. Research indicates that older adults who maintain high levels of social activity and robust social networks, especially those engaging in regular physical activities, exhibit lower risks of obesity and diabetes (23, 24). Furthermore, urban dynamics such as the heat island effect and dense population distributions can precipitate extreme weather conditions, posing direct threats to the health of older adults—a demographically vulnerable group (25). The urban pace, competitive pressures, and high living costs, coupled with the downsizing and nuclearization of family structures, may culminate in social isolation and loneliness among older adults, potentially deepening depressive symptoms and severely impairing their self-care capabilities and overall health.

Although a number of studies have explored urban and geriatric health (see Table 1), there are still gaps to be addressed. (1) Many studies have focused on the health impacts of urbanization or urban sprawl but have not concentrated on mental health. (2) Although some studies have explored the impacts of the urbanization process on depression, there is no literature confirming whether the planning of urban agglomerations has a direct association with depressed mood. (3) The implementation of policies has not been studied by analyzing their impacts on depression in specific populations. To bridge these gaps and thoroughly assess the influence of urban agglomeration planning on the mental health of older adults, this study employs an empirical analysis using the multi-temporal double-difference-in-differences (DID) model, leveraging data from the China Health and Retirement Longitudinal Study (CHARLS) and regional economic indicators from the China Statistical Yearbook. The findings are further substantiated through a series of robustness tests. Additionally, this research investigates the heterogeneous impacts of urban agglomeration planning on geriatric depression across different demographics, categorized by gender, region, and educational level, providing new insights and recommendations for a more nuanced assessment and implementation.

**Table 1:** Literature review on the impact of cities on health.

Studies	Research topics	Population	Main findings
Song et al. (81) 2024	Urban health advantage and penalty in aging populations	Older adults	Public facilities and economic activities contribute to health and may offset the negative impacts of reduced green space and increased air pollution.
Zhang et al. (82) 2024	Health impacts of fine particulate matter shift due to urbanization	No specific population	Air pollutants cause premature deaths during urbanization.
Polemiti et al. (83) 2024	How does the macroenvironment influence brain and health	No specific population	A comprehensive overview of the impact of the macro-environment on mental illness.
Wang et al. (32) 2024	The health and welfare effects of environmental governance	People aged 45 and over	The health of middle-aged and older adults has been significantly enhanced by reducing air pollution and improving social capital.
Xu et al. (26) 2023	Effects of urban living environments on mental health in adults	Adults	Exposure to complex environments in urban life was positively associated with affective symptoms, and green space was negatively associated with anxiety symptoms.
Dong et al. (66) 2021	The impact of carbon emissions on residents’ health	No specific population	Higher health risks in highly industrialized and urbanized areas
Wang et al. (84) 2021	Health threats from extreme urban heat	Older adults	Compound urban heat poses a threat to healthy women and older urban residents
Astell et al. (27) 2019	Associations between urban green spaces and adult mental health	Adults	Exposure to more total green space was associated with lower rates of psychological distress.
Lu et al. (85) 2019	The role of urbanization in aggravating the health burden	No specific population	Mortality densities are much higher in densely populated urban areas than in rural areas.

Compared to previous studies, this research makes three contributions: (1) Theoretically, it provides a new perspective for studying the factors influencing older adult depression and the impact of urban agglomeration planning on the health sector. (2) Practically, through a quantitative analysis framework, it integrates urban agglomeration planning with the issue of older adult depression, offering targeted recommendations and strategies. (3) Methodologically, the DID model was applied to effectively identify the policy effects of urban agglomeration planning, thereby partially mitigating endogeneity issues.

## 3 Impact mechanisms and research hypotheses

**Figure 1:**
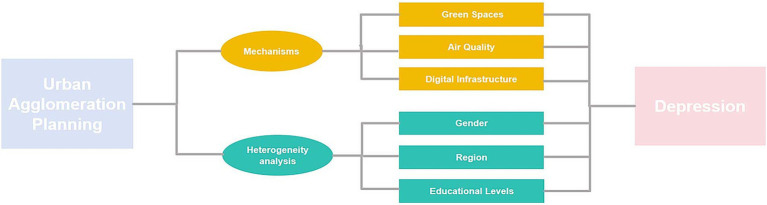
The framework diagram on the impact of urban agglomeration planning on depression.

Based on depression-related influencing factors and the impact of cities on the health of the older adult, from the perspective of mechanism analysis, the planning of urban agglomerations affects the mental health of older adults mainly through three ways: increasing the area of green space, improving air quality, and developing infrastructure construction. At the same time, we comprehensively considered heterogeneity analysis based on factors such as gender, region, and education level to explore differences in the responses of older people from different social groups in the implementation of the policy, ensuring that the results of the study are generalizable and have stronger explanatory power (see Figure 1).

### 3.1 Direct effects of urban agglomeration planning on depression in older adults

Urban agglomeration planning, as a comprehensive policy system, aims to harmonize resource allocation and development strategies within a region to enhance the overall quality of life. For older adults, urban agglomeration planning may directly affect their mental health status. First, the implementation of such planning promotes regional economic development and enhances the provision of public services—including healthcare, older adult care, and social support—thus better meeting the basic needs of older adults. Second, urban agglomeration planning optimizes the urban environment, improves residential comfort and safety, and reduces the stress and anxiety experienced by older adults (26). In addition, by strengthening community building and promoting social interaction, urban agglomeration planning helps to reduce loneliness and social isolation among older adults (10). All of these direct improvements help to reduce the incidence of depression among the older adult. Based on this, the following hypothesis is proposed in this study:

*Hypothesis 1:* Urban agglomeration planning helps reduce the incidence of depression among the older adult.

### 3.2 Mechanistic assumptions

#### 3.2.1 Urban green space

An important goal of urban agglomeration planning is to increase urban green spaces and open spaces through rational spatial layouts and environmental protection measures. These green spaces not only enhance the quality of residents’ living environments but also help relieve the psychological pressure of the older adult and reduce the incidence of depression (27). Based on this, this paper explores how increasing urban green space affects the mental health of the older adult from the following three aspects. First, exposure to the natural environment is widely recognized as having a positive effect on mood regulation. Increasing urban green spaces and natural areas provides older adults with a safe and tranquil environment that helps alleviate life stress and reduce anxiety and depressive symptoms (28). Second, urban green spaces provide places for older people to engage in outdoor activities and social interactions. Green spaces are ideal locations for older people to engage in light exercises such as walking and Tai Chi, as well as to socialize and interact with others. Increased outdoor activity helps improve older adults’ physical health, prevent chronic diseases such as obesity and cardiovascular disease, and enhance cognitive function (29). Social enrichment, on the other hand, significantly reduces loneliness and enhances social support among older adults, which is especially critical for alleviating depressive symptoms (24). Finally, improved physical health and increased social interactions further enhance the psychological resilience and well-being of older adults, forming a positive feedback loop between physical and psychological health. In summary, urban agglomeration planning systematically enhances the physical and mental health of older adults through multiple pathways—including environment, socialization, and health—by increasing urban green spaces and open spaces. Based on the above analysis, this study proposes the following hypothesis:

*Hypothesis 2:* Urban agglomeration planning contributes to reducing the risk of depression among the older adult by increasing urban green space.

#### 3.2.2 Air quality

Air pollution is an important risk factor for the mental health of older adults, especially long-term exposure to pollutants such as PM2.5 and sulfur dioxide, which can trigger chronic inflammation, impair brain function, and increase the risk of depression (30). Therefore, improving air quality is crucial for maintaining the mental health of older people and reducing the risk of depression. Urban agglomeration planning has significantly reduced air pollutant emissions by promoting clean energy, reducing reliance on highly polluting energy sources, and optimizing public transportation systems. These measures have been effective in improving air quality, reducing the incidence of respiratory and cardiovascular diseases, and enhancing the physical health of older people (22). Improved physical health can alleviate the psychological burden caused by chronic diseases among the older adult, reduce feelings of helplessness and despair, and lower the risk of depression. In addition, better air quality creates a more favorable environment for seniors to participate in outdoor activities. Unlike the natural spaces provided by increased urban green areas, improved air quality removes barriers that previously prevented older people from engaging in outdoor activities due to health concerns. More opportunities for outdoor activities help enhance social interactions and community participation, reducing loneliness and social isolation among older adults (10). Based on the above analysis, the following hypothesis is proposed in this study:

*Hypothesis 3:* Urban agglomeration planning contributes to reducing the risk of depression among older adults by improving air quality.

#### 3.2.3 Infrastructure development

Urban agglomeration planning positively impacts the mental health of older adults through improved infrastructure development, particularly by enhancing access to the Internet and digital technologies. Enhancing digital infrastructure enables older adults to integrate more fully into society, access information, and enjoy various services, thereby reducing the risk of depression (31). First, the widespread availability of the Internet has expanded the social networks of older adults. With the help of digital platforms, older adults can stay in close contact with their families, friends, and communities, reducing social isolation and loneliness. This enhanced social interaction and emotional support helps alleviate depressive symptoms and improve mental health (18). Second, digital services have enhanced the accessibility of medical and mental health support for older adults. Through telemedicine and online consultations, older adults can access professional medical services in a timely manner, especially those with limited mobility or living in remote areas. This facilitates early detection and intervention of depression and improves treatment outcomes. In addition, the Internet provides a platform for older adults to learn and acquire health knowledge. Through online education and health applications, older adults can improve their health literacy, enhance their self-management abilities, and prevent depression. In summary, Internet infrastructure development within urban agglomeration planning improves the mental health of older adults in multiple dimensions by promoting social integration, enhancing healthcare accessibility, and supporting self-health management. Based on the above analysis, this study proposes the following hypothesis:

*Hypothesis 4:* Urban agglomeration planning contributes to reducing the risk of depression among older adults by promoting the development of Internet infrastructure.

## 4 Empirical research design

### 4.1 Empirical model

In recent years, the impact of urban policies on public health has attracted much attention. Using a difference-in-differences (DID) model, Wang et al. (32) explored the impact of environmental policies on human health and overall well-being, emphasizing the important role of policy interventions in improving residents’ health. Similarly, Liu et al. (33) utilized the DID model to examine the impact of smart city pilot policies on the social adaptation and mental health of middle-aged and older adult people. Given that urban agglomeration planning, as a policy piloted in batches and gradually rolled out, possesses characteristics of a quasi-natural experiment, a quasi-experimental design is appropriate to assess its impact. Drawing on these methods, this study applies a multi-period DID model to systematically analyze the impact of urban agglomeration planning on the depressive symptoms of the older adult, taking into account the timing and regions of the urban agglomeration planning pilots. Specifically, the following model is constructed in this paper:


$$\text{Sdepressio}n_{\text{it}}=\alpha_{0}+\alpha_{1}DID_{\text{it}}+\alpha_{2}\text{Contro}l_{\text{it}}+\gamma_{i}+\mu_{t}+\varepsilon_{\text{it}}$$

In the assessment of the intervention’s effectiveness, the DID model mitigates the impact of confounding variables on experimental outcomes by incorporating both the “before and after difference” and the “difference between the conditions before and after planning implementation.” To address potential discrepancies between the experimental and control groups, the DID model incorporates covariates that account for influencing factors. This adjustment compensates for the inherent limitations of sample allocation in natural experiments, which cannot be entirely randomized, thereby enhancing comparability between the groups (34). Consequently, this study employs the DID model to empirically validate and investigate the effects of urban agglomeration planning on depression among older adults.

In this model, *Sdepression* is measured by depressive symptoms in older adults, with subscript i indicating the individual senior, and t denoting the year. In the DID framework, older adults are assigned a value of 1 if they resided in the area targeted for urban agglomeration planning in year t, and 0 otherwise; this assignment corresponds to the interaction term typically used in traditional DID models. Depression in older adults is affected by a combination of factors, which can be considered at the level of personal characteristics, socio-economic and so on. Studies have shown that from the viewpoint of personal characteristics, factors such as gender (35), education level (36), smoking and drinking (37), health status (Health status is a comprehensive assessment of an individual’s current level of health, measured using a self-assessed health score) (38), and living alone (39) all have an impact on depression. In terms of urban macroeconomic variables, economic level (40), urbanization rate (41), population density (42), and urban industrial structure (43) affect the subjective well-being of the older adult. Based on the influencing factors of depression and concerning Wang et al.’s study (44), this paper selects individual characteristics variables and urban macroeconomic variables as the control variables in this paper. The individual characteristics of older adults include gender (*gender*), education level (*edu*), marital status (*married*), health score (*srh*), whether they smoke (*smoke*), and whether they drink (*drink*). Macroeconomics and urban variables include *annual income*, the logarithm of GDP (Gross Domestic Product) growth rate (*lngdp*), the logarithm of GDP *per capita* (*lnmgdp*), the number of people per million square kilometers (*lnmidu*), the logarithm of the urbanization rate (*lnurban*), and logarithm of industrial structure (*lnindus*). This study controls for regional fixed effects $\gamma_{i}$ and annual fixed effects $\mu_{t}$. The coefficient $\alpha_{1}$ measures the effect of the urban agglomeration planning’s implementation and reflects the net impact of these policies on depression among older adults. Fixed effects are utilized throughout the study to control for unobserved heterogeneity within the data, ensuring that the estimated effects are attributed solely to the planning intervention and not to external, uncontrolled factors.

### 4.2 Construction of indicators

#### 4.2.1 Symptoms of depression in older adults

In the CHARLS database, the prevalence of depression among older adults was evaluated using the CES-D-10 scale, a well-validated instrument comprising eight negatively phrased items and two positively phrased items (45). The negative items include symptoms such as “feeling annoyed,” “experiencing concentration difficulties,” “feeling frustrated,” “perceiving that everything requires effort,” “feeling fearful,” “experiencing sleep disturbances,” “could not get going,” and “feeling lonely.” The positive affect items are “feeling hopeful” and “feeling happy.” Responses to each item are quantified based on frequency, with options ranging from “rarely or not at all” to “most or all of the time.” Scoring for negative items is assigned from 0 to 3, correlating directly with the increasing frequency of symptoms, whereas scoring for positive items is inversely calculated, with higher scores reflecting less frequent positive emotions. The aggregate score for an individual can range from 0 to 30, where a higher score indicates more severe depressive symptoms and an elevated risk of depression. Based on existing literature, a cutoff score of 10 on the CES-D-10 is utilized to differentiate between high-risk and low-risk depression, with scores of 10 or higher indicating a high risk of depression. The reliability and validity of the CES-D-10 scale in this study are robust, evidenced by a Cronbach’s alpha of 0.799 and a Kaiser-Meyer-Olkin (KMO) measure of 0.889, indicating good internal consistency and sampling adequacy for the analysis.

#### 4.2.2 Urban agglomeration planning

**Figure 2:**
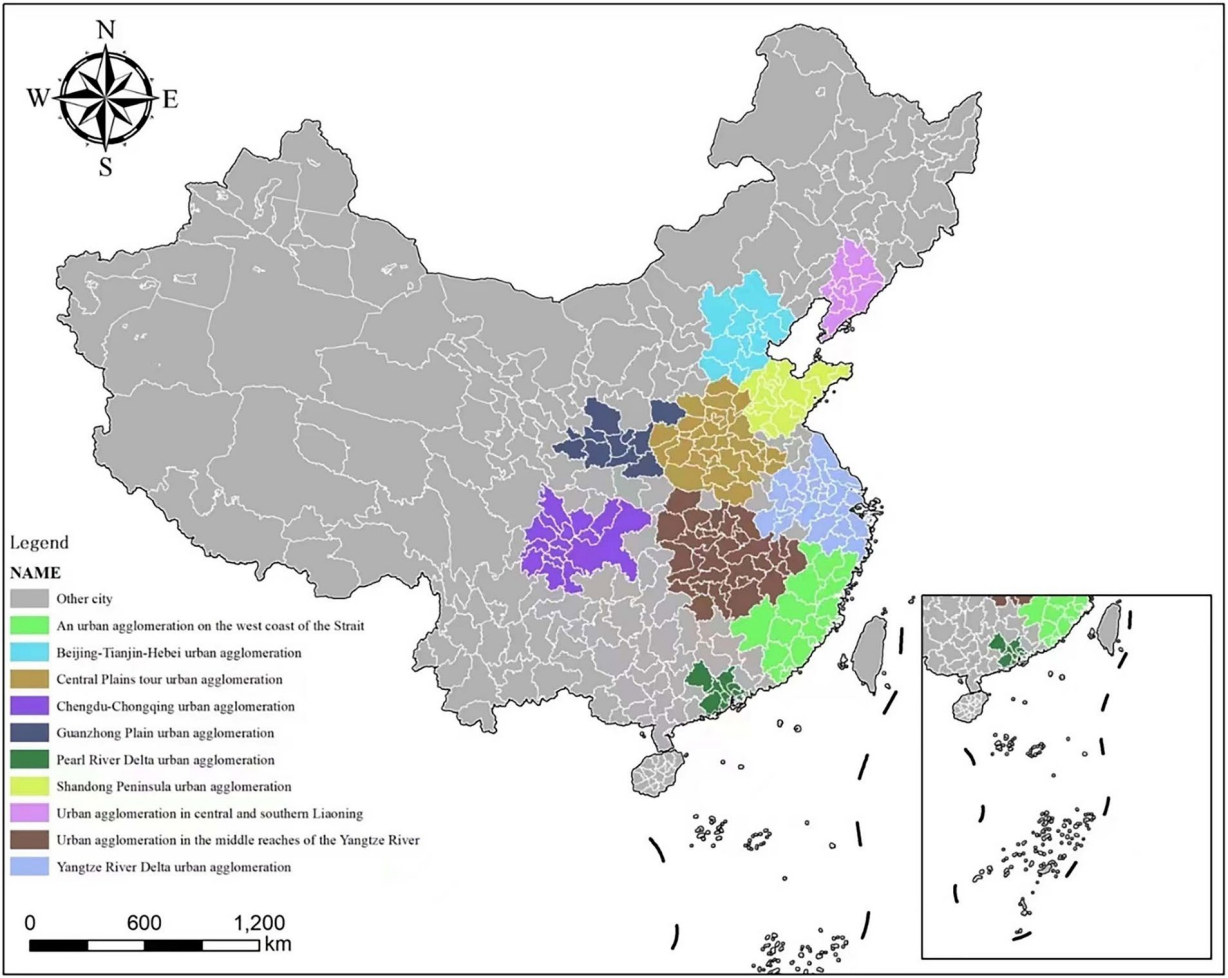
Pilot progress of the urban agglomeration planning in China.

Urban agglomeration planning refers to the organic integration of geographically adjacent or functionally complementary cities into a networked structure within a specific region through scientific and systematic planning (46). Usually centered around a core city as the hub and relying on the linkage and cooperation of surrounding node cities, it forms a multi-center and multi-level urban system. The aim is to optimize the allocation of regional resources, promote synergistic cooperation among cities, maximize the economic, social, and ecological benefits of the region, and achieve regional integration and sustainable development. In the context of rapid urbanization, urban agglomeration planning has attracted much attention as a key strategy for shaping urban development patterns and optimizing the urban living environment to promote higher-quality and sustainable city development (47, 48). This move not only affects the economic prosperity and social progress of cities, but also directly influences the quality of life and health of the residents (49). Based on the plans for the development of 19 urban agglomerations published on the central government’s website of the People’s Republic of China, the urban agglomeration plans were used as the central explanatory variable in this study to explore their impact on depression in older adults. To distinguish between older adult populations in areas where urban agglomeration planning has been implemented and those in areas where it has not, we set the dummy variable for older adult residents in implemented areas to 1 and those in non-implemented areas to 0 (50). Figure 2 illustrates the designated regions included in the urban agglomeration planning pilot.

### 4.3 Data description

The data for this study were sourced from the CHARLS database, complemented by regional economic variables obtained from the China Statistical Yearbook. Descriptive statistics of the variables used are summarized in Table 2. The CHARLS database, a large-scale interdisciplinary survey project, is sponsored by the Institute of National Studies at Peking University and administered by the China Social Science Research Center. This survey uniquely addresses the multidimensional aspects of aging in China, combining comprehensive assessments of older adults with the specific demographic characteristics of the nation. CHARLS employs a rigorous multistage, random sampling methodology. Initially, 150 districts and counties were selected based on a probability proportional to size sampling technique, accounting for regional distribution, urban–rural classification, and *per capita* income levels. In the selected 150 districts and counties, a total of 450 village-level units were chosen using random sampling, with 3 units selected from each district or county. In each selected household, one individual aged 45 years or older was randomly chosen as the primary respondent, and their spouse was also included in the sample. Since its inception in 2011, CHARLS has successfully conducted baseline data collection across 28 provinces, autonomous regions, and municipalities directly under the central government. Follow-up surveys were carried out in 2013, 2015, and 2018, with the longitudinal cohort study concluding in 2018. By the end of the 2018 survey wave, the database comprised a sample of 19,000 participants from 12,400 households. For this analysis, data spanning from the 2011 inception of the database through to 2018 were utilized. Samples with missing data were excluded, and continuous variables were adjusted by trimming the top and bottom 1% to mitigate the effects of outliers. The resultant dataset for this study comprises a balanced panel of 45,603 observations. The Biomedical Ethics Review Committee of Peking University approved the CHARLS study (approval number IRB00001052–11015), and all interviewees were required to provide informed consent.

**Table 2:** Variable description and summary statistics.

Variables	Description	Obs	Mean	Standard deviation	Min	Max
Sdepression	/	45,603	8.19	6.122	0	30
did	/	45,603	0.283	0.451	0	1
Household variables						
gender	The gender of the respondent: Male = l; Female = 0	45,603	0.472	0.499	0	1
edu	The education level of the respondent: Education levels below primary school =1; Primary education = 2; Junior high school education =3; Educational level of high school or above =4	45,603	2.044	1.049	1	4
married	Marital status of the respondent: Not married = 0; Married = 1	45,603	0.889	0.314	0	1
Health behaviors						
smoke	Whether the respondent smokes: Not smoke = 0; Smoke = 1	45,603	0.267	0.442	0	1
drink	Whether the respondent drinks: Not drink = 0; Drink = 1	45,603	0.352	0.478	0	1
srh	From the CHARLS survey, a higher score suggests the respondent perceives themselves as healthier: Very poor = 1 Poor = 2 Fair = 3 Good = 4 Very good = 5	45,603	2.924	0.986	1	5
Provincial variables						
annual income	Annual income in years	45,603	14889.175	22926.983	0	1,212,387
lngdp	Logarithm of GDP growth rate	45,603	1.93	0.671	−2.996	3.027
lnmgdp	Logarithm of GDP *per capita*	45,603	10.64	0.582	8.842	13.056
lnmidu	Persons per million square kilometers	45,603	510.163	294.606	8.9	2348.27
lnurban	Logarithm of the urbanization	45,603	3.962	0.252	3.161	4.615
lnindus	Logarithm of industrial structure	45,603	0.628	0.039	0.486	0.767

## 5 Empirical results and analysis

### 5.1 Benchmark regression results

This study examines the effect of urban agglomeration planning on depression in older adults. The model regression results are in Table 3. Column (1) of Table 3 gives the estimation results without any control variables, individual and year effects. The DID coefficients are significantly negative at the 1% level indicating that the urban agglomeration planning reduces depression among older adults. In column (2), the variables of individual characteristics of older adults and macroeconomic and social variables are added, including health scores (*srh*), education level (*edu*), gender (*gender*), marital status (*married*), whether they smoke (*smoke*), and whether they drink alcohol (*drink*), annual income (*annual income*), the logarithm of GDP growth rate (*lngdp*), logarithm of GDP *per capita* (*lnmgdp*), number of people per million square kilometers (*lnmidu*), logarithm of the urbanization rate (*lnurban*), logarithm of the industrial structure (*lnindus*). The regression results show that although the DID coefficient is still negatively significant at the 1% level, the model’s goodness of fit increases, further illustrating the stability of the benchmark regression.

**Table 3:** Benchmark regression results.

	(1)	(2)
	Sdepression	Sdepression
did	−0.4573***	−0.3931***
	(0.1114)	(0.1014)
srh		2.2738***
		(0.0260)
edu		−0.7090***
		(0.0266)
married		−1.6284***
		(0.0798)
smoke		0.1844***
		(0.0685)
drink		−0.0287
		(0.0592)
gender		−1.4675***
		(0.0651)
annual income		0.0000
		(0.0000)
lngdp		0.2362***
		(0.0742)
lnmgdp		0.1055
		(0.2522)
lnmidu		0.0001
		(0.0004)
lnurban		−1.0658
		(0.6909)
lnindus		−15.5182***
		(3.3420)
_cons	8.3196***	17.5207***
	(0.0420)	(3.1005)
Observations	45,603	45,603
*R*-squared	0.072	0.260

Standard errors are in parentheses, *** *p* < 0.01, *** denote significance at the 1% levels, and the numbers in parentheses are robust standard errors clustered by firms.

### 5.2 Heterogeneity test

#### 5.2.1 Gender

The gender disparity in depression is notably pronounced, with women experiencing a higher incidence and greater susceptibility to depression compared to men. According to a study published in The Lancet, the burden of mental disorders among women surpasses that of men across all geographical regions, with the global prevalence of depression being 64.8% higher in women than in men (51). This disparity suggests that the effects of urban agglomeration planning on depression could be more pronounced among female urban residents. The empirical analysis detailed in Table 4 corroborates this hypothesis. Columns (1, 2) present the regression results for female and male subjects, respectively. As shown in column (1), the findings for females reveal a coefficient of −0.6085, which is statistically significant at the 1% confidence level. In contrast, the coefficient for males, presented in column (2), is −0.1329 and does not reach statistical significance at the 10% confidence level. This indicates a substantial mitigative effect of urban agglomeration planning on depression among women.

**Table 4:** Heterogeneity of gender.

	Female	Male
	(1)	(2)
	Sdepression	Sdepression
did	−0.6085***	−0.1329
	(0.1470)	(0.1377)
srh	2.5070***	2.0047***
	(0.0377)	(0.0354)
edu	−0.8533***	−0.5811***
	(0.0406)	(0.0349)
married	−1.5565***	−1.7257***
	(0.1079)	(0.1196)
smoke	0.7076***	0.1490**
	(0.2079)	(0.0692)
drink	0.1365	−0.1813**
	(0.1020)	(0.0705)
annual income	−0.0000	0.0000
	(0.0000)	(0.0000)
lngdp	0.2836***	0.1624
	(0.1064)	(0.1020)
lnmgdp	0.2322	0.0718
	(0.3572)	(0.3530)
lnmidu	−0.0001	0.0003
	(0.0006)	(0.0005)
lnurban	−1.2071	−0.5235
	(0.9970)	(0.9433)
lnindus	−13.1476***	−19.0716***
	(4.8365)	(4.5428)
_cons	14.7500***	17.1603***
	(4.4564)	(4.2634)
observations	24,059	21,544
*R*-squared	0.259	0.222

Standard errors are in parentheses, *** *p* < 0.01, ** *p* < 0.05, and * *p* < 0.1.

#### 5.2.2 Region

The implementation of urban agglomeration planning in China exhibits significant geographic specificity, with distinct policy priorities in the western, central, and eastern regions. The western region, predominantly characterized by resource-based industries such as resource development, energy, chemicals, and metallurgy, experiences considerable environmental impact. Resource extraction activities often result in substantial energy consumption and emissions, releasing significant amounts of pollutants including sulfur dioxide, nitrogen oxides, and particulate matter. Research has indicated that prolonged exposure to such polluted environments can engender feelings of powerlessness, frustration, and hopelessness, thereby elevating the risk of depressive moods (52). Consequently, the implementation of urban agglomeration planning may have a heterogeneous impact on residents’ mental health due to these geographic distinctions. Columns (1, 2, 3) in Table 5 present the empirical regression results for older adult samples from the Western, Central, and Eastern regions, respectively. Column (1) shows the regression results for the Western region, with a coefficient of −0.6717, significant at the 1% confidence level; column (2) displays the results for the Central region, with a coefficient of −0.5052, also significant at the 1% confidence level. Column (3) represents the Eastern region, where the coefficient is not significant at the 10% confidence level. These findings indicate that urban agglomeration planning has a more pronounced impact on reducing depression among older adults in the Western and Central regions.

**Table 5:** Heterogeneity of region.

	Western region	Central region	Eastern region
	(1)	(2)	(3)
	Sdepression	Sdepression	Sdepression
did	−0.6717***	−0.5052***	−0.1871
	(0.2426)	(0.1696)	(0.1903)
srh	2.4642***	2.3587***	2.0339***
	(0.0507)	(0.0433)	(0.0419)
edu	−0.8619***	−0.7098***	−0.5986***
	(0.0530)	(0.0427)	(0.0442)
married	−1.6690***	−1.4790***	−1.7504***
	(0.1472)	(0.1343)	(0.1334)
smoke	−0.0129	0.1979*	0.3920***
	(0.1325)	(0.1127)	(0.1129)
drink	0.1668	−0.0109	−0.2517**
	(0.1103)	(0.0980)	(0.0998)
gender	−1.6915***	−1.5753***	−1.1467***
	(0.1264)	(0.1064)	(0.1079)
annual income	−0.0000*	0.0000	0.0000***
	(0.0000)	(0.0000)	(0.0000)
lngdp	0.1284	0.1056	0.4370**
	(0.5077)	(0.5218)	(0.4392)
lnmgdp	0.4638	−0.5439	−0.3293
	(0.5077)	(0.5218)	(0.4392)
lnmidu	−0.0023	0.0006	−0.0017
	(0.0019)	(0.0005)	(0.0016)
lnurban	−2.2092*	−2.7872*	1.7473
	(1.2283)	(1.4463)	(1.5330)
lnindus	−22.5678***	−12.2488**	−9.8916
	(6.5373)	(5.0144)	(9.4148)
_cons	23.6827***	28.9558***	7.8470
	(7.1539)	(5.4807)	(8.5807)
observations	13,835	16,324	15,444
*R*-squared	0.261	0.254	0.230

Standard errors are in parentheses. *** *p* < 0.01, ** *p* < 0.05, and * *p* < 0.1.

#### 5.2.3 Educational levels

Depression is a multifaceted condition influenced by an array of factors including genetics, environmental exposures, social support networks, personal coping mechanisms, and educational levels. Existing research indicates that lower educational levels are associated with a heightened vulnerability to depression (53). This study, therefore, investigates the potential heterogeneity in the effects of urban agglomeration planning on the depressive status among individuals with varying educational backgrounds. The empirical findings from this analysis are detailed in Table 6, which segregates the sample of older adults based on their educational attainment: education levels below primary school, primary education, junior high school education, and educational level of high school or above. The regression results are as follows: Column (1): The regression coefficient for older adults with less than primary education is −0.5292, significant at the 1% confidence level. Column (2): For individuals with primary education, the coefficient is −0.4537, significant at the 5% confidence level. Column (3): For those with junior high school education, the coefficient is −0.2022, which is not significant at the 10% confidence level. Column (4): The regression for individuals with more than a high school education shows a coefficient of −0.0185, which remains statistically insignificant at the 10% confidence level. These findings reveal that the influence of urban agglomeration planning on depression is more pronounced among older adults with lower levels of education, specifically those with less than primary or only primary education.

**Table 6:** Heterogeneity of educational levels.

	Education levels below primary school	Primary education	Junior high school education	Educational level of high school or above
	(1)	(2)	(3)	(4)
	Sdepression	Sdepression	Sdepression	Sdepression
did	−0.5292***	−0.4537**	−0.2022	−0.0185
	(0.1719)	(0.1957)	(0.2074)	(0.2534)
srh	2.3622***	2.2806***	2.1095***	2.0771***
	(0.0424)	(0.0510)	(0.0533)	(0.0710)
married	−1.4543***	−1.8241***	−1.7950***	−1.8090***
	(0.1151)	(0.1718)	(0.1958)	(0.2476)
smoke	0.0868	0.1687	0.3342***	0.3161**
	(0.1324)	(0.1267)	(0.1285)	(0.1547)
drink	−0.0175	0.0323	−0.0040	−0.3096**
	(0.1065)	(0.1119)	(0.1168)	(0.1377)
gender	−1.7161***	−1.4224***	−1.5235***	−0.6792***
	(0.1186)	(0.1232)	(0.1298)	(0.1559)
annual income	0.0000	0.0000	0.0000	0.0000
	(0.0000)	(0.0000)	(0.0000)	(0.0000)
lngdp	0.2377	0.2476*	0.1882	0.1967
	(0.1445)	(0.1450)	(0.1263)	(0.1723)
lnmgdp	−0.2255	0.2732	0.4239	−0.2219
	(0.4262)	(0.5032)	(0.5139)	(0.6172)
lnmidu	−0.0002	0.0005	0.0002	0.0011
	(0.0008)	(0.0008)	(0.0007)	(0.0008)
lnurban	−2.0311*	1.5516	−0.2692	−2.9970
	(1.0732)	(1.4293)	(1.4844)	(1.9174)
lnindus	−11.7052**	−27.9413***	−17.6021***	−7.1870
	(5.6362)	(6.8959)	(6.4501)	(8.1535)
_cons	21.6115***	11.4952*	10.7649*	20.5496**
	(5.0224)	(6.3702)	(6.5233)	(8.1381)
observations	18,653	11,775	9,706	5,469
*R*-squared	0.234	0.237	0.225	0.217

Standard errors are in parentheses. *** *p* < 0.01, ** *p* < 0.05, and * *p* < 0.1.

### 5.3 Robustness tests

To test the robustness of the previous empirical results, the study performs the following robustness tests.

#### 5.3.1 Parallel trend test

**Figure 3:**
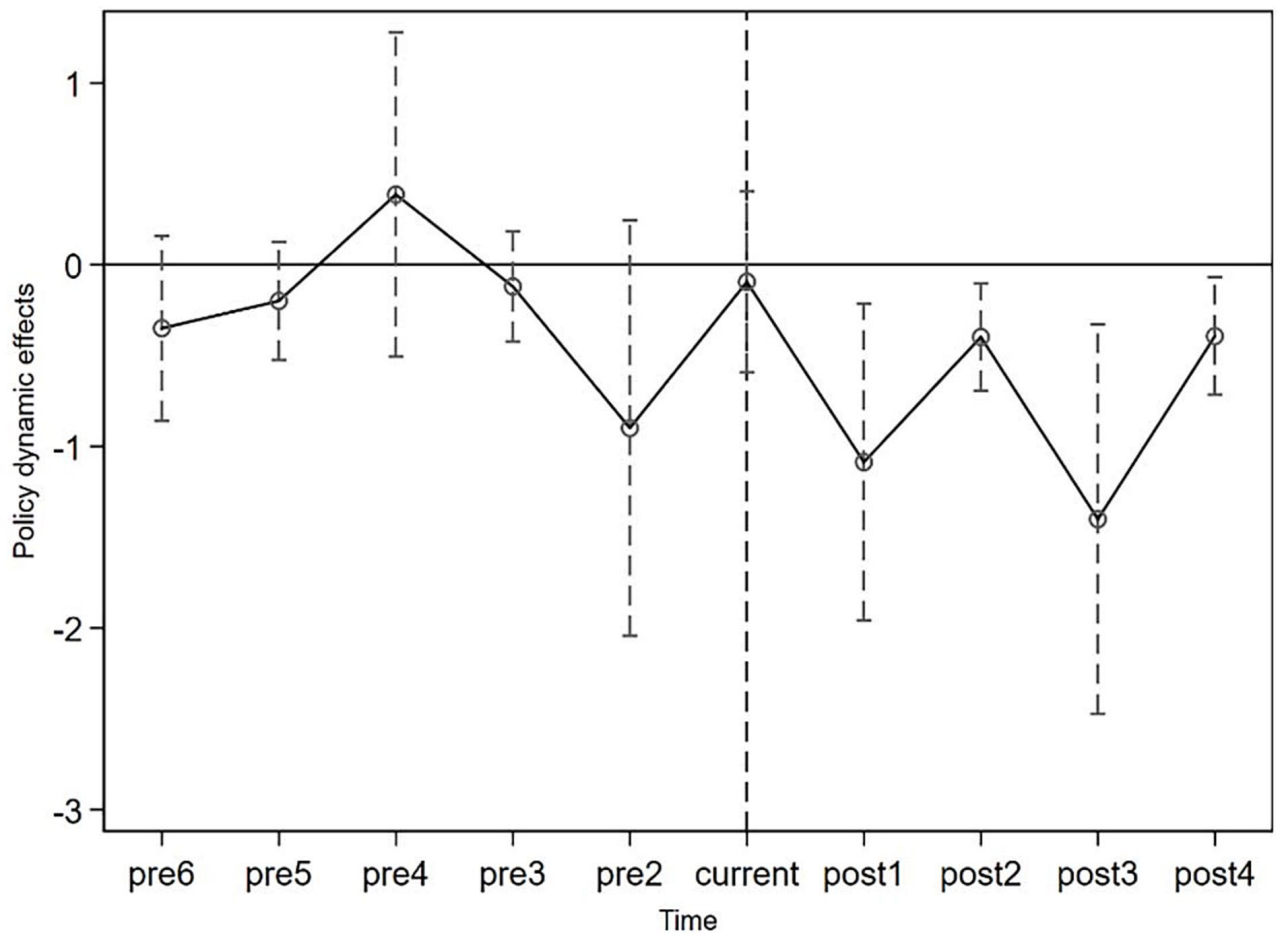
Parallel trend test.

The DID methodology fundamentally relies on the assumption that parallel trends exist between the treatment and control groups before the implementation of the plan. Deviations from this assumption could lead to either overestimating or underestimating the effect. To validate the assumption of parallel trends, our analysis identifies the year in which the urban agglomeration planning was initially implemented as the baseline. The analysis spans the effects of 6 years before the planning implementation and 4 years afterwards. Figure 3 illustrates the results of the parallel trend test. Figure 3 indicates that, before the implementation of the urban agglomeration planning, the impact on older adult depression was not significantly different from zero. From a dynamic perspective, the effect is observed from the first to the fourth year after implementation, reflecting a sustained accumulation of the impact. This demonstrates that the planning has a robust and enduring effect on alleviating depression among older adults, signifying that the parallel trend test has been successfully passed.

#### 5.3.2 Placebo test for exclusion of randomized outcomes

**Figure 4:**
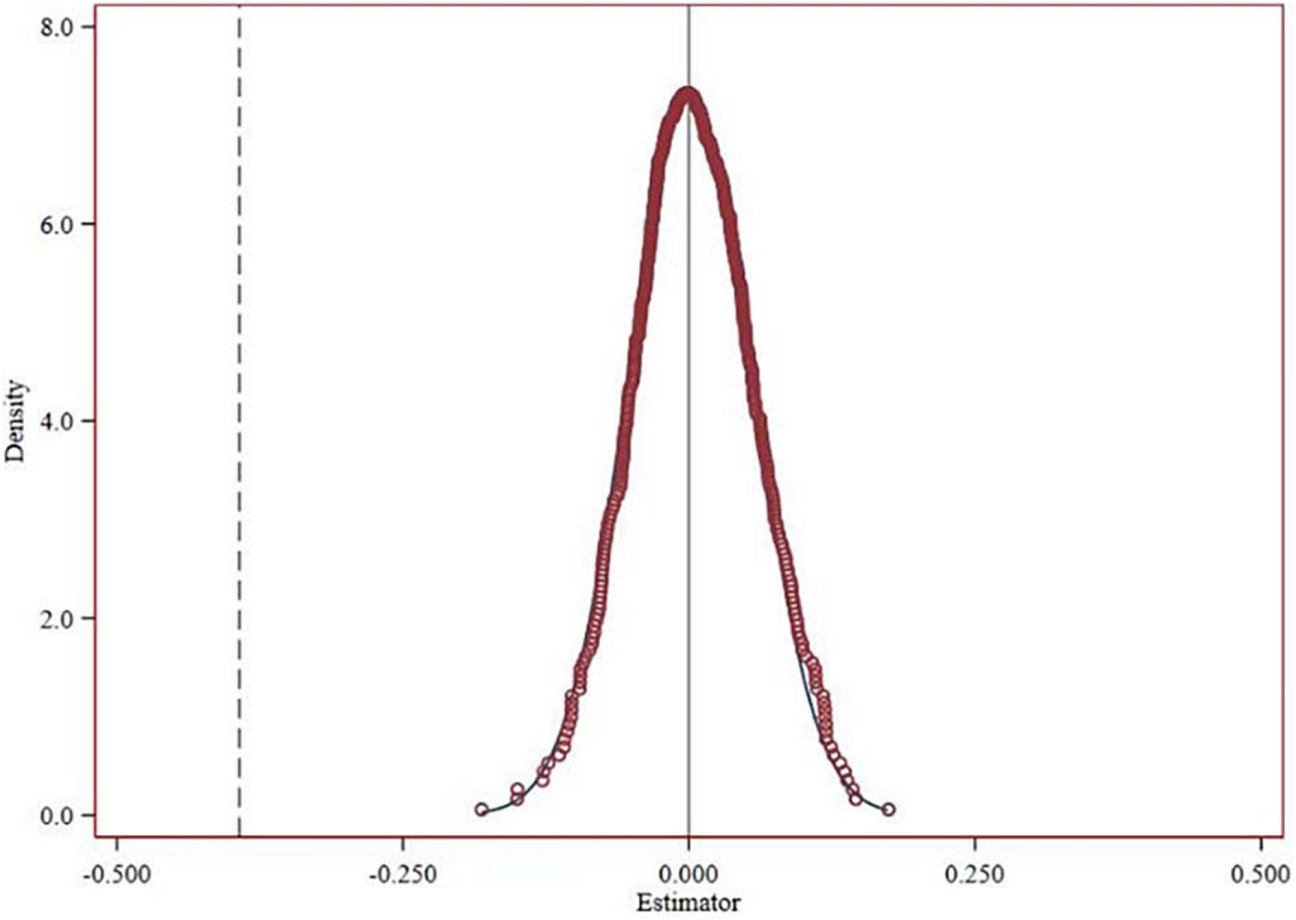
Placebo test chart.

To mitigate the potential influence of random variations, this study adopted a methodology inspired by Chetty et al. (54) in which the implementation years and regions for the urban agglomeration planning were randomized. This procedure was conducted 1,000 times in a placebo trial to ensure robustness. The outcomes, illustrated in Figure 4, demonstrate that the distribution of regression coefficients from the randomization simulation clusters around zero, whereas the coefficients from the benchmark regression are distinctly separate from this distribution. These findings indicate that the empirical results of this study are not influenced by random factors.

#### 5.3.3 PSM-DID

To mitigate self-selection bias, this study employed a propensity score matching double difference (PSM-DID) model that integrates the propensity score matching (PSM) and DID model. This approach was utilized to validate the robustness of our regression outcomes and to mitigate bias resulting from potentially unobserved factors. The control variables were used as the matching criterion, and the sample was derived using the 1:1 nearest neighbor matching technique (55). The outcomes are presented in Table 7. These regression results delineate the impact of urban agglomeration planning on depression among older adults. The use of nearest-neighbor matching serves to somewhat alleviate the problem of sample self-selection, thereby reducing bias in the estimation of the DID model. The findings remain statistically significant at the 1% level, confirming that the baseline regression results are robust.

**Table 7:** PSM-DID test results.

	Sdepression
did	−0.3926***
	(0.1041)
srh	2.2730***
	(0.0264)
edu	−0.7141***
	(0.0271)
married	−1.6477***
	(0.0811)
smoke	0.1909***
	(0.0696)
drink	−0.0372
	(0.0602)
gender	−1.4694***
	(0.0661)
hhcperc	0.0000
	(0.0000)
lngdp	0.2408***
	(0.0748)
lnmgdp	0.0799
	(0.2608)
lnmidu	0.0001
	(0.0004)
lnurban	−0.5721
	(0.7314)
lnindus	−16.1436***
	(3.4160)
_cons	16.2404***
	(3.2833)
observations	44,103
*R*-squared	0.260

Standard errors are in parentheses. *** *p* < 0.01.

### 5.4 Mechanism tests

According to the results of the baseline regression, urban agglomeration planning can help alleviate depressive symptoms in older adults. This study will continue to test the mechanism through theoretical analysis. The combination of policies and existing literature indicates that the construction of green space in urban agglomeration planning should ensure sufficient area and multifunctionality, improve accessibility and ecological connectivity, and adopt eco-friendly design to enhance the quality of life of residents (56). At the same time, urban agglomeration planning promotes the transformation of the energy structure, increases the use of clean energy, promotes green buildings and energy-saving technologies, reduces the consumption of high-polluting energy, improves air quality, promotes the sustainable development of the city, and enhance the mental health and quality of life of residents (46). Additionally, the construction of digital infrastructure in urban agglomeration planning is crucial for the development of modernized and intelligent cities. Efficient, convenient, safe, and sustainable development of urban agglomerations is achieved through high-speed broadband, wireless networks, data centers, and cloud computing. Through high-speed networks and smart devices, the older adult can conveniently conduct remote medical consultations and treatment to reduce the psychological pressure caused by health problems, thus effectively promoting the mental health of the older adult (57). Based on these analyses, this study argues that urban agglomeration planning alleviates the level of depression in older adults through a series of mechanisms, and we investigate the mechanisms of urban agglomeration planning’s effect on depression in the older adults mediated by green space area, air pollution, and digital infrastructure development.

#### 5.4.1 Green space area

This study examined the impact of urban agglomeration planning on green space *per capita* using green space *per capita* as a mediating variable. The results of the mechanistic analysis of green space area *per capita* are presented in column (1) of Table 8. The results show that urban agglomeration planning significantly increased the green space area *per capita* and improved the depression of the older adult in the region.

**Table 8:** Mechanism analysis.

	Green space area	Air pollution	Digital infrastructure development	
	(1)	(2)	(3)	(4)
Explained variables	Green space *per capita*	Number of days with air quality at or better than level 2	The logarithm of the number of Internet broadband subscribers (Ten thousand households)	The logarithm of the year-end number of cell phone subscribers (Ten thousand households)
did	0.7820^***^	7.3662^***^	0.0495^***^	0.0495^***^
	(0.0634)	(0.9895)	(0.0053)	(0.0053)
lngdp	0.2988^***^	−222.6281^***^	−0.0723^***^	−0.0723^***^
	(0.0556)	(7.8013)	(0.0038)	(0.0038)
lnmidu	−0.0064^***^	−36.1519	0.0003^***^	0.0003^***^
	(0.0003)	(31.0741)	(0.0000)	(0.0000)
lnurban	−3.1826^***^	−8.2798^***^	0.6547^***^	0.6547^***^
	(0.4924)	(0.6510)	(0.0347)	(0.0347)
lnurban	15.7197^***^	0.1199^***^	3.7829^***^	3.7829^***^
	(2.2537)	(0.0102)	(0.1623)	(0.1623)
_cons	18.7246^***^	1132.1096^***^	1.0782^***^	1.0782^***^
	(1.9425)	(35.1922)	(0.1456)	(0.1456)
Observations	35,245	31,937	45,603	45,603
*R*-squared	0.699	0.772	0.993	0.993

Standard errors in parentheses, * *p* < 0.10, ** *p* < 0.05, *** *p* < 0.01.

#### 5.4.2 Air pollution

Considering that air pollution is influenced by airflow, the number of days with air quality at and better than Class II was used as a proxy variable for air pollution in this study given the availability of data. Column (2) of Table 8 presents the results of air pollution mechanism analysis. It shows the regression results in a 1% level at which these coefficients are significantly positive. The results indicate that urban agglomeration planning significantly enhances air quality and positively impacts the population’s mental health.

#### 5.4.3 Digital infrastructure development

Internet broadband and mobile telephony are core components of the digital infrastructure that provide basic communications and Internet access. Based on this this study uses the logarithm of the number of Internet broadband subscribers (Ten thousand households) and the logarithm of the number of cell phone subscribers at the end of the year (Ten thousand households) as proxy variables. Columns (3, 4) of Table 8 display the results of the analysis of the mechanisms behind digital infrastructure development. The results show that after the implementation of urban agglomeration planning, there is a significant positive contribution to alleviating the level of depression among the older adult with the increase of digital facilities.

## 6 Discussion

### 6.1 Discussion of baseline regression results

Our benchmark regression results substantiate that urban agglomeration planning has significantly improved depression levels among older adults. This finding not only aligns with the macro-policy objectives of global development but also echoes China’s national development strategy to build inclusive, safe, and sustainable urban environments that provide accessible public spaces and support services for older groups (11). Our findings reiterate the perspective of Carsten Butsch et al., suggesting that urban agglomeration planning can influence population health (58). The difference, however, lies in our study’s focus on mental health, specifically extended to older adults. A potential explanation for this result is that, before the implementation of the urban agglomeration planning, the urban population was long exposed to high concentrations of ozone (59), which is significantly associated with mortality, respiratory diseases, and cardiovascular diseases (60). Furthermore, residents along the Yangtze River basin have long been afflicted by water pollution. It is estimated that approximately 45,804,500 tons of untreated industrial wastewater are discharged directly into the Yangtze annually, adversely affecting the health of older adult residents, including their mental well-being (61). Additionally, as urban populations increase rapidly, the urban layout becomes denser, potentially limiting adequate space for physical activity for the older and diminishing their opportunities for social interaction, which can negatively impact their mental health (62). The implementation of urban agglomeration planning has effectively improved the lifestyles of older residents. On the one hand, this planning may have enhanced environmental quality, reducing the exposure of older adults to harmful conditions. For example, it may have implemented measures to reduce the emissions of harmful substances such as ozone and water pollution, thereby lowering the risk of respiratory and cardiovascular diseases among the older adult. These environmental improvements likely contribute positively to the health of older adults, thereby benefiting their mental health. On the other hand, urban agglomeration planning can promote social interaction among older adults by increasing opportunities for social and recreational activities. Planning might support the development of more public transport infrastructure, parks, recreational facilities, and community centers, offering older adults greater opportunities to participate in group activities. Such social interactions help to mitigate feelings of loneliness and social isolation among the older adult, enhancing their psychological well-being and life satisfaction. Thus, the implementation of urban agglomeration planning has positively impacted the mental health of older adults, particularly by improving environmental quality and fostering social interaction.

### 6.2 Heterogeneity effect analysis

#### 6.2.1 Gender heterogeneity effect analysis

**Figure 5:**
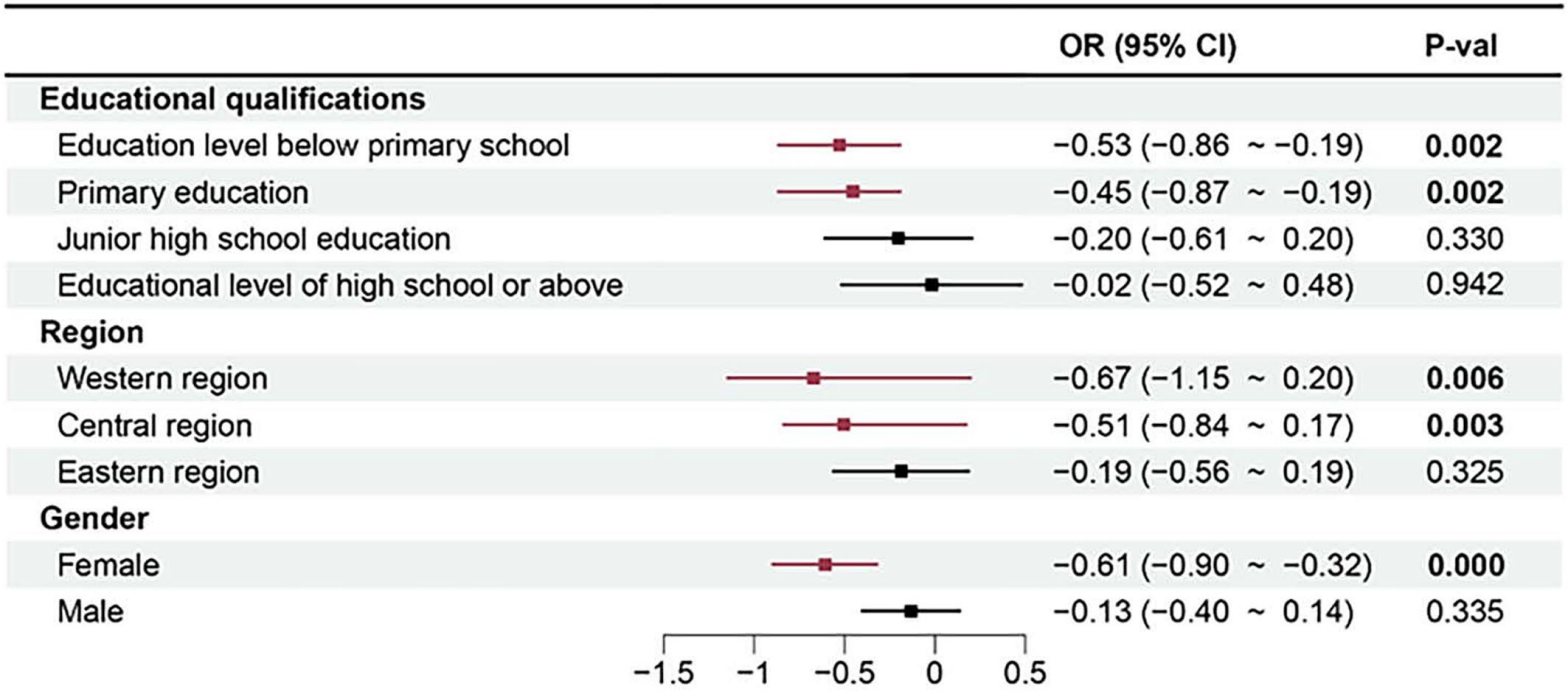
Heterogeneity analysis forest plot.

To visualize the results of the heterogeneity distribution more visually, we depicted a forest plot of the heterogeneity analysis (see Figure 5).

In the figure, red lines represent statistically significant results, while black lines represent non-significant results. The results in column (1) of Table 4 are significant at the 1% confidence level, whereas the coefficients in column (2) are not significant. The forest plot in Figure 5 shows the same outcomes, further suggesting that urban agglomeration planning has a more pronounced impact on depression among women. Urban agglomeration planning may significantly impact women by improving social and urban environments. On the one hand, older adult women typically rely more on social networks and community support to maintain mental health than their male counterparts (63). Urban agglomeration planning that facilitates transportation and increases social interactions is particularly vital for older adult women, who are more likely to benefit from social engagement with others. On the other hand, older adult women are more concerned with the quality of their external environment (64). Urban agglomeration planning often includes measures to improve environmental quality, such as reducing pollutant emissions and creating more green spaces. These environmental improvements may have a more significant impact on the physical and mental health of older adult women. In addition, Urban older adult women, due to high life pressure, social isolation, and environmental pollution, are at greater risk of depression. In contrast, limited mental health resources in rural areas may lead to an underestimation of depressive symptoms among rural women (5). Future research could further compare and analyze the mental health status of women in urban areas with that of women in rural areas, and explore in depth whether there are urban–rural differences in depression symptoms among women as a result of urban agglomeration development.

#### 6.2.2 Regional heterogeneity effect analysis

The heterogeneity effect analysis from Figure 5 indicates that urban agglomeration planning has a more pronounced impact on depression among older adults in the western and central regions. The eastern region of China typically has better resource endowments and economic conditions compared to the central and western regions (65). Economically, the eastern region of China is more mature and diversified compared to the central and western regions, suggesting that older adults in the central and western regions may face greater economic pressures and social inequalities. The urban agglomeration planning may have significantly alleviated these pressures by increasing employment opportunities and strengthening social security, thereby having a more pronounced impact on the mental health of the older adult in the central and western regions. Additionally, infrastructure development tends to lag in the central and western regions compared to the eastern region. There may be disparities in medical facilities, cultural and recreational facilities, and community activity venues between the central and western regions and the eastern region. Urban agglomeration planning could promote infrastructure development in the central and western regions, providing more conveniences and support, which is beneficial for the mental health of older adults. However, this study currently analyses the country by dividing it into three regions—western, central, and eastern. While this division may present general trends among the regions, it may not fully capture the spatial variability within each region. Indeed, urban agglomerations within regions differ significantly from non-urbanized areas in terms of geographic, economic, and social conditions, such as pollution levels, industrial layouts, degrees of economic development, and distributions of healthcare resources (66). These factors may exhibit greater heterogeneity within the same region, thereby affecting the mental health of older adults to different degrees. Additionally, there are differences in economic and industrial layouts within city clusters. Some cities have actively promoted industrial transformation and upgrading, improving the environment and quality of life, while others still rely on traditional industries, which may lead to differences in the living environment and mental health status of the older adult. Therefore, specific impacts at smaller scales may be overlooked if these regions are treated only as a single unit of analysis.

#### 6.2.3 Educational level heterogeneity effect analysis

Columns (1, 2, 3, 4) of Table 6 display the regression results for older adult samples with education levels below primary school, primary education, junior high school education, and educational level of high school or above, respectively. These results indicate that urban agglomeration planning has a more pronounced impact on depression among older adult individuals with lower education levels. This is consistent with the heterogeneity test results shown in Figure 5. The reasons may lie in the differences in educational levels, which can lead to variations among older adult in their needs for social support, their capacity to access and understand information, and their economic circumstances. Firstly, older adult individuals with lower literacy levels often rely more heavily on social support to maintain their mental health. Urban agglomeration planning can enhance this support by providing more community activities and services for older adults. These resources can help reduce loneliness and increase social interactions, thereby improving mental health (67). Secondly, older adults with lower literacy levels may find it more challenging to access and understand health-related information and are less likely to engage in proactive mental health behaviors (68). Urban agglomeration planning can help bridge this information gap by providing more widespread and accessible health promotion and education, enabling those with lower literacy to benefit more from the positive impacts. Thirdly, the older adult with lower literacy levels often have lower economic incomes and social status, which correlates with greater life stress and social inequality (69). Urban agglomeration planning might directly benefit economically disadvantaged older adults by improving social security measures, thereby exerting a more substantial impact on their mental health.

### 6.3 Discussion of mechanism test results

Based on the validation of the inhibitory effect of urban agglomeration planning on the mental health of older adults, this study further explores the possible influence mechanisms of the above effects. First, urban green spaces are essential mechanisms through which urban agglomeration planning affects the mental health of older adults. Urban agglomeration planning policies often establish clear policies and regulations to ensure that a certain percentage of green space must be included in new urban developments (70). Numerous studies have shown that urban green space is negatively associated with depression in older adults (71, 72) and that green space not only reduces anxiety in older adults by providing fresh air and beautiful landscapes but also provides recreational and leisure venues that enhance older adults’ interactions with others (73). On the other hand, green space can mitigate the urban heat island effect and create a more pleasant temperature environment (28, 74). Second, air pollution is also an important mechanism. Urban agglomeration planning policy not only promotes economic development but also enforces environmental regulations in urban planning, thereby improving air quality (75, 76). For older adults, a better air environment can improve their sleep quality (77), reduce their risk of cardiovascular and respiratory diseases (78), and increase their satisfaction and sense of security in their living environment, all of which can contribute to older adult’s mental health. Third, urban agglomeration planning policies promote Internet development, which in turn affects the mental health of older adults. Network infrastructure development is an important part of urban agglomeration planning. In urban agglomeration planning, governments and related agencies often prioritize the development of digital infrastructure, investing in high-speed broadband technologies (fiber optics, 5G, etc.) to improve Internet coverage and speed throughout the region (79). On the one hand, good Internet services can ensure that the older adult can more easily stay in touch with their families and access online recreational resources, which will help reduce loneliness and enhance psychological well-being (80). On the other hand, there are numerous mental health resources and online counseling services available on the Internet through which older adults can access emotional support and mental health advice.

## 7 Conclusions and implications

### 7.1 Conclusion

Analysis using parallel trend tests, propensity score matching (PSM), and placebo tests reveals that urban agglomeration planning significantly influences the depression status of older adults. Heterogeneity test analyses indicate that the effects of urban agglomeration planning on depression are more pronounced among older women, older adults in the central and western regions, and those with lower education levels.

### 7.2 Policy recommendations

These findings offer a solid empirical base for further investigation into the construction of urban agglomerations to improve the mental health of older adults. Based on this evidence, we propose the following policy recommendations. To accelerate the adoption of successful urban agglomeration experiences, it is crucial to create a livable environment. This can be achieved by increasing urban greenery, improving air quality, optimizing transportation, and enhancing infrastructure along with community medical services. These initiatives can significantly reduce stress and anxiety levels among residents. At the same time, it is essential to strengthen social care and support services tailored for older adults. This can be accomplished by maintaining a variety of social activities for older adults through dedicated activity centers, volunteer programs, and social gatherings, and by establishing a comprehensive network of health and psychological counseling services. Such measures are instrumental in enhancing the quality of life and overall well-being of the older adult.

We also recommend developing differentiated urban agglomeration planning. The improvement in depression among older adults due to urban agglomeration planning exhibits heterogeneity. Policy implementation must consider objective conditions such as geographic location and educational levels. Given the disparities in resource allocation, industrial structures, and living environment quality across different regions, depression levels among older adults vary. The pronounced positive impact of urban agglomeration planning on mental health in the western and central regions highlights the importance of targeted policy implementation. Therefore, the government should increase resource allocation to further enhance the quality of life and mental health of older adults in these regions.

Lastly, based on the research findings, future policies, and social services should focus more on older adult individuals with lower education levels, providing them with increased social support, financial assistance, and mental health services to help them better adapt to the changes brought about by urban agglomeration development.

### 7.3 Limitations

Firstly, while the CHARLS database offers a comprehensive array of health and living condition questionnaires, it may not encompass all variables that influence health outcomes. Secondly, our study primarily aimed to illuminate the impact of urban agglomeration planning on depression among older adults. However, we did not explore potential mediating effects, such as moderation or multichain mediation, which could provide deeper insights into the dynamics at play. Future studies should consider employing structural equation modeling. This approach would allow researchers to more comprehensively analyze the relationships and effect sizes of potential mediating variables, thereby offering a more nuanced understanding of how various factors interact to influence the mental health of older adults within the context of urban agglomeration policies.

## Data Availability

The original contributions presented in the study are included in the article/supplementary material, further inquiries can be directed to the corresponding authors.
