# Corrigendum: The impact of urban agglomeration planning on depression in older adults

**DOI:** 10.3389/fpubh.2025.1607430

**Published:** 2025-04-23

**Authors:** Ya Liu, Li Yan, Yujue Wang, Xiaotang Tang, Ming Gao, Jiayu Yang, Zuoyan Liu, Xiuying Hu

**Affiliations:** ^1^Innovation Center of Nursing Research and Nursing Key Laboratory of Sichuan Province, West China Hospital, Sichuan University/West China School of Nursing, Sichuan University, Chengdu, Sichuan, China; ^2^Department of Critical Care Medicine, West China Hospital, Sichuan University, Chengdu, Sichuan, China; ^3^China Academy of Engineering Physics, Mianyang, Sichuan, China; ^4^Sichuan Vocational College of Commerce, Chengdu, Sichuan, China; ^5^School of Economics, Sichuan University, Chengdu, Sichuan, China; ^6^School of Business, Chengdu University of Technology, Chengdu, Sichuan, China; ^7^Department of Rehabilitation Medical Center, West China Hospital, West China School of Nursing, Sichuan University, Chengdu, Sichuan, China

**Keywords:** urban agglomeration planning, older adults, depression, difference-in-differences model, CHARLS

In the published article, there was an error in affiliation1. Instead of “Innovation Center of Nursing Research and Nursing Key Laboratory of Sichuan Province, West China Hospital, Sichuan University, West China School of Nursing, Sichuan University, Chengdu, Sichuan, China”, it should be “Innovation Center of Nursing Research and Nursing Key Laboratory of Sichuan Province, West China Hospital, Sichuan University/West China School of Nursing, Sichuan University, Chengdu, Sichuan, China”.

The authors apologize for this error and state that this does not change the scientific conclusions of the article in any way. The original article has been updated.

